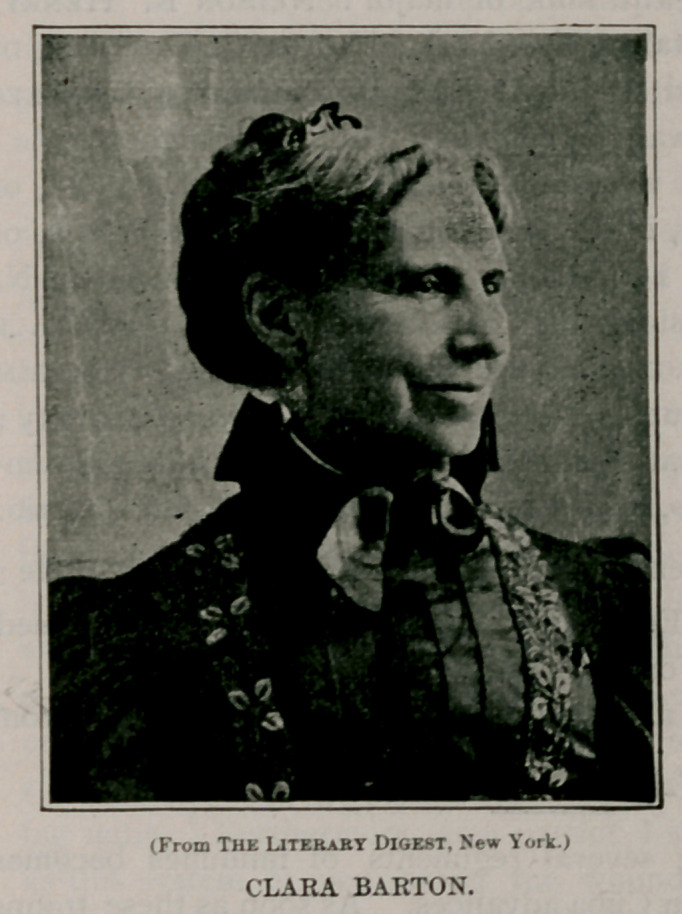# Army and Navy Notes

**Published:** 1898-08

**Authors:** 


### ARMY AND NAVY NOTES.

SURGEON-GENERAL STERNBERG has requested through the adjutant-general's office that an investigation be made of the charges that the transport Seneca, which brought sick and wounded from General Shafter's army at Santiago to New York, left the former place with an inadequacy of medical supplies and was otherwise unfit for the work assigned to her. From letters which have been received by General Sternberg it appears that specially unfavorable weather conditions prevailed and the surf was running high when the ship was being loaded with her human freight, though why a sufficient quantity of medical supplies was not put on board the vessel is not explained. There was plenty of quinine and also plenty of morphine available with the medical corps, but it appears that not enough of the latter drug was sent aboard the vessel for all purposes necessary and on this account some of the complaints have arisen. The Seneca was not designed for a hospital ship and consequently there may have been some lack of necessary facilities for caring for the sick and wounded.

It is probable that much complaint originated from the fact that civilians and foreign military attachés were given passage on the Seneca. Miss Jeanette Jennings, the Red Cross nurse, appears to have rendered excellent service and to have kept her head level; so, too, did Captain Dowdy, commandant of the ship. The praises of both were sounded by the sick and wounded that the ship brought safely into port.

---

BRIGADIER-GENERAL LEONARD WOOD, United States Volunteers, was until recently in command of the first Volunteer Cavalry, known as "Roosevelt's Rough Riders." He is an intimate friend of Colonel Roosevelt and it was the latter who urged his appointment to the command.

He is the only officer of the medical staff thus far during the present war who has risen to the rank of brigadier-general; hence a sketch of his life may prove of interest.

General Wood was born in New Hampshire and was appointed to the army from Massachusetts. In 1886 he became past assistant surgeon, with the rank of captain, and was assigned to duty with the 8th Infantry, then stationed in the Southwest. When Geronimo's raid began Captain Wood took command of a detachment of the regiment and pursued the Indian band, finally capturing the leader and his followers and taking them into Texas. For his services in this campaign and in that of 1888 he received an honor medal. Duty in California and in Georgia followed. He was then stationed in Washington and on the death of Dr. Newton L. Bates, surgeon-general of the navy, who was serving as the president's physician, Dr. Wood became his successor as physician at the White House.

The plan of organising a regiment of plainsmen was Captain Wood's. He saw the advantages which men trained to outdoor life and hardships would have over other recruits. The idea met with the approval of Secretary Alger and of Mr. Roosevelt, at that time assistant secretary of the navy. How well Colonel Wood's views were borne out by the heroic fight of the Rough Riders at La Quasina is a matter of too recent history to need more than a passing mention. On July 8th President McKinley sent Colonel Wood's name to the Senate to be a brigadier-general, and the Senate promptly ratified the appointment. General Wheeler also recommended him for galantry in action.

General Wood has succeeded General McKibben as military commandant at Santiago, the latter having become disqualified by ill-health. General Wood's appointment is most opportune, his knowledge of sanitary science coming into special play at this time. He has thoroughly policed the city, buried the dead animals that were everywhere giving forth noisome stenches and is otherwise putting it into cleanly condition.

---

THAT the Red Cross has received official recognition by the government is indicated by the following letter :

WAR DEPARTMENT,
WASHINGTON, June 6, 1898.

(From THE LITERARY DIGEST, New York.)

CLARA BARTON.

MISS CLARA BARTON, *President American National Red Cross, Washington, D. C.:*

DEAR MADAM—The tender of the services of the American National Red Cross, made to this department through the department of state, under date of May 25, 1898, for medical and hospital work—as auxiliary to the hospital service of the army of the United States—is accepted, all representatives and employes of said organisation to be subject to orders according to the rules and discipline of war, as provided by the Sixty-third Article of war.

Very truly yours,

R. A. ALGER,
*Secretary of War.*

This settled a moot question that threatened some bitterness and gives the Red Cross official standing. Miss Barton has been doing good service in the Santiago campaign. Her ship, the State of Texas, was loaded with supplies in advance and sailed with General Shafter's fleet. Red Cross nurses and supplies were opportune as soon as the fighting commenced and became a material support to the medical department. The Texas was the first ship to enter Santiago harbor after the surrender and the distribution of its supplies ameliorated hunger and distress incident to the siege.

---

A HOSPITAL ship for Manilla is preparing under the auspices of the Red Cross of Oakland, Cal., which is contemplating the purchase of a hospital ship to be sent to the Phillipines. It is estimated to cost about $300,000. The national society, it is reported, will sanction the plan.

---

THE following appointments have been announced by the war department since our July issue:

To be chief surgeon, with rank of lieutenant-colonel, Major PHILIP F. HARVEY, surgeon U. S. A.

To be division surgeons, with rank of major: NELSON R. HENRY, assistant surgeon-general of New York; VICTOR C. VAUGHAN, of Michigan, surgeon 33d Michigan Volunteer Infantry; CHARLES M. ROBERTSON, of Iowa, surgeon Iowa Volunteer Infantry.

To be brigade surgeons, with rank of major: ROYCE DAY FRY, of Ohio; ELMER E. HEG, of Washington; CHARLES R. PARKE, of Pennsylvania, surgeon 13th Pennsylvania Volunteers, JAMES N. JACKSON, of Missouri, surgeon 3d Missouri Volunteers; WALLACE HEFF, of Ohio; GEORGE F. SHIELDS, of California; WILLIAM S. BRYANT, assistant surgeon 1st Massachusetts Heavy Artillery; WILLIAM F. DENIEDMAN, assistant surgeon 22d Kansas Volunteers; FRANCIS C. FORD, of Texas; LAWRENCE C. CARR, of Ohio.

The following assignments of Buffalo physicians have been made:

Acting Assistant Surgeon EDWARD J. MEYER, U. S. A., will proceed from Washington to Fort Monroe, Virginia, for duty.

Acting Assistant Surgeon IRA C. BROWN, U. S. A., will proceed from Washington to Tampa, Florida, for assignment to duty.

---

THE wisdom of enlisting several regiments of immunes becomes apparent as the campaign in Cuba advances. As soon as these troops reach Santiago it will enable General Wood to garrison the city with soldiers absolutely safe from the infection of yellow fever, thus enabling the present garrison to return to the hills where there is fresh air and good water. This is another way in which modern sanitary science contributes to the art of war. Whoever heard of a proposition from Spain to enlist immunes or to contribute in any way to the arrest of infectious disease in Cuba? On the contrary, it has been guilty for centuries of offending the civilisation of the world by its maintenance of unsanitary surroundings at Havana, through which yellow fever has been distributed hither and yon wherever sailors went or ships touched. Let the end of such misrule come speedily and even, if need be, with vengeance!

---

THERE has been considerable agitation over the pension question since the beginning of the present war. Some pensioners have feared their allowances would cease, others that they would be diminished, while still others have advocated surrendering their certificates from patriotic motives to help meet the war expense. In this relation we think the following anecdote of General John A. Dix deserves publication as indicating a high order of patriotism, for it is a complete answer to those who are eternally carping at the pensioners. The time was in the civil war. The General was seen in line before a pension agency by an intimate friend, who took him to task somewhat after this fashion:

"General, I am ashamed to see you here. You, rich, receiving a large salary as commander of this district and in the hour of your country's distress drawing a pension and you do not need it!"

The General responded: "I am glad that you have spoken so freely, sir. Every word of that is true. I am rich. I do receive a large salary. My country is in distress for money and I do not need the pension. But I am here to draw my pension because I am entitled to it. I served my country in a previous war and was wounded in that service. The understanding has always been in this country that in war the soldier should not be paid full wages. But afterward, for wounds or for incapacity incurred from that service he should be paid a pension. That, sir, was the agreement, just as it has been in all our wars, and in order that no poor soldier shall be ashamed to stand up for his pension I am here to give him countenance by drawing mine. Furthermore, as a patriot I speak. This nation, so long as this system of pensioning the wounded soldier lasts, shall never want for soldiers; nor shall they lack the fighting spirit."

---

SURGEON-GENERAL STERNBERG proposes to have the much discussed question as to the utility of the so-called cholera bands settled by official action. At his instance a board of medical officers, to consist of Colonel Dallas Bache, assistant-surgeon general; Lieutenant-Colonel Charles Smart, deputy-surgeon general and Major Walter

Reed, surgeon, is appointed to meet in Washington as soon as practicable for the purpose of considering the question as to whether or not the government should issue the so-called "cholera bands" for the use of United States troops in the field.

Meanwhile patriotic women all over the land are busily at work manufacturing these belts. Should the board decide the matter favorably a large supply probably will be shipped at once to the troops in the tropics.

The immediate needs of the soldiers, however, are intelligently set forth by Colonel J. Morris Brown, in charge of medical supplies at the army building in New York, who recently said that there was no use in sending nightshirts to the men. What they wanted was pajamas, which could be used in the field as well as in the hospital. A suggestion made lately was that safety pins would be of much more use to soldiers than ordinary pins, cushions full of which have been contributed for their use. One of the prime necessities of a soldier is tobacco and in nine cases out of ten nothing is more highly appreciated.

A check for $50 lately has been forwarded to Colonel Brown by the Hartford Soldiers' Aid Society, for use in purchasing delicacies for the wounded troops. Good use can be made of all such gifts, which may be sent to him at the Army Building, No. 39 Whitehall street, New York.

---

ANOTHER hospital ship is fitting out for the use of the army. The former Atlantic Transport Line steamer Missouri, now at pier No. 19, East river, New York, has been selected for this service. The work was begun before the Missouri left Philadelphia a few days ago, when seventy men were placed on board of her and the work was continued throughout her trip to New York. The men, with an additional force, will work night and day until the ship is made ready for this important duty. When the changes are completed she will be able to carry 600 wounded men and her equipment will be of the latest design.

The auxiliary societies of the Red Cross have given to the ship an ice plant, a carbonising plant, a steam laundry, a steriliser and a steam launch, all of the value of $25,000. The ship will also have distilling apparatus, as well as *x*-ray and other modern scientific equipments. The government has ordered that the Missouri be ready for active duty by August 10th, and it is expected that she will then proceed to Porto Rico.

If Mr. Richard Harding Davis "and valet" would take to the woods and not try to dictate as to the general conduct of the army in Cuba, including its medical department, it would be at least better for himself if not for his "valet." The criticisms he offered of the management of the campaign before Santiago by General Shafter in his dispatch of July 6th, and of the medical department in that of July 7th, should at least have rendered him *ingrata* and have caused his expulsion from the lines. As he has not been heard from since the last mentioned date, it is presumed that General Shafter has exercised his prerogative in this respect. The sooner the newspaper correspondents are taught that they cannot invade the secret councils of the army, nor write in criticism of the officers with the same careless indifference to personal character that they exhibit toward individuals in civil life, the better for them and for the world at large.

---

Surgeon-General Sternberg of the army has received numerous letters from patriotic women asking what articles would be most acceptable for the use of our sick and wounded soldiers in the field or in the hospitals. For the purpose of answering in a general way these inquiries he has prepared the following memorandum which will be sent to persons communicating with him on this subject:

Money may be sent to the surgeon-general of the army as a contribution to the hospital fund of the hospital ship Relief and of the United States general hospitals. This will be sent to the surgeons in charge to be expended for delicacies for the sick, such as canned soups, jellies, lemons, oranges and the like. Those who prefer may contribute canned soups, clam broth, orange marmalade, ginger ale, biscuits, water crackers and similar articles in hermetically sealed cans for use on the hospital ship Relief and at the United States general hospitals at Key West, Fla., Fort McPherson, Ga., Fort Thomas, Ky., and Fort Myer, Va. Bandages, lint and other surgical dressings are not desired, as these can be obtained from the manufacturers sterilised for use and of the quality which experience has shown to be best suited to our purposes.

Shirts and drawers are provided by the government, but will be accepted and can be given to convalescents upon their discharge from hospital. Pajamas, made of light gingham, will be useful for the sick in hospital and on the hospital ship, as they can be worn by convalescents who are able to be out of bed. Long night shirts of light muslin can also be utilised. Broad bandages of light flannel, to protect the abdomen, are highly recommended and may be worn to advantage by our soldiers in the field.

The articles mentioned may be sent direct to the surgeon-general of the army at Washington or to the surgeons in charge of the general hospitals named.

---

"The battle of July third," which has become the official designation of the naval tournament off Santiago de Cuba in which Admiral Sampson's fleet destroyed the Spanish squadron under Admiral Cervera, was remarkable in that the American loss was but one man killed and twenty-two wounded, most of the latter being slight injuries from splinters or fragments of shells. They were brought north by the Solace and are progressing favorably under the influence of good air and excellent attention. Admiral Cervera, defeated though he was, has borne himself with dignity. He is about the only Spaniard in public life, from Sagasta down to the humblest orderly, that is entitled to the respect of mankind. For the rest, they are a bragging, deceptive, treacherous lot, while Eulate, captain of the Vizcaya, has even worse manners, if it were possible, than Mayor Van Wyck. But Admiral Cervera by his whole bearing shows himself to be a kind-hearted, brave and cultured gentleman.

## Figures and Tables

**Figure f1:**